# The impact of reducing dietary aflatoxin exposure on child linear growth: a cluster randomised controlled trial in Kenya

**DOI:** 10.1136/bmjgh-2018-000983

**Published:** 2018-12-01

**Authors:** Vivian Hoffmann, Kelly Jones, Jef L Leroy

**Affiliations:** 1 Markets, Trade and Institutions Division, International Food Policy Research Institute (IFPRI), Washington, DC, USA; 2 Economics Department, American University, Washington, DC, USA; 3 Poverty, Health and Nutrition Division, International Food Policy Research Institute, Washington, District of Columbia, USA

**Keywords:** aflatoxin, child linear growth, serum aflatoxin level, exposure, Kenya

## Abstract

**Introduction:**

Observational studies have documented an association between aflatoxin (AF) exposure and reduced linear growth in infants and young children. Our objective was to assess the effectiveness of reducing AF exposure on child linear growth and serum AF levels in rural areas in Eastern Kenya.

**Methods:**

A cluster randomised controlled design was used (28 intervention and 28 control clusters). The intervention arm received a swapping (contaminated maize was replaced with safe maize) and a stockist intervention (households were encouraged to purchase from a stockist supplied with clean maize). Women in the fifth to final month of pregnancy were invited to enrol in the study. Outcomes were child length-for-age Z-score (LAZ), the prevalence of stunting and child serum AFB_1_-lysine adduct level 24 (endline, primary outcomes) and 11 to 19 months (midline, secondary outcomes) after trial commencement, respectively. The trial was registered with socialscienceregistry.org.

**Results:**

Of the 1230 unborn children enrolled in the study, 881 (72%) were included in the LAZ and 798 (65%) in the serum AFB_1_ analysis. The intervention significantly reduced endline ln serum AFB_1_-lysine adduct levels (intervention effect—0.273, 95% CI −0.547 to 0.001; one-sided p=0.025), but had no effect on endline LAZ or stunting (mean LAZ at endline was −1.64). At midline, the intervention increased LAZ by 0.16 (95% CI −0.009 to 0.33; one-sided p=0.032) and reduced stunting by seven percentage points (95% CI −0.125 to −0.007; one-sided p=0.015), but had no impact on serum AFB_1_ levels.

**Conclusion:**

Improving access to AF-free maize substantially reduced endline serum AF, but had no effect on child linear growth. The midline analysis suggests that AF may affect linear growth at younger ages.

**Trial registration number:**

AEARCTR-0000105.

**Key questions**

**What is already known?**

- Aflatoxin B1 (AFB1) produced by *A. flavus* and *A. parasiticus*, can lead to death from aflatoxicosis when consumed in high doses and is a potent carcinogen.
- Observational studies showing an association between AFB1 exposure and reduced linear growth in utero and in young children cannot fully exclude confounding.

**What are the new findings?**

- This study is the first randomised controlled design to examine the impact of reducing aflatoxin exposure on child linear growth and serum aflatoxin levels.
- The intervention significantly and substantially reduced serum aflatoxin levels, but had no effect on child linear growth at endline (primary outcomes).

**What do the new findings imply?**

- The midline effect on linear growth (secondary outcome) suggests that future studies should explore these age-varying effects.

## Introduction

Aflatoxins (AFs) are a group of naturally occurring mycotoxins that pose important health risks. *Aspergillus flavus* and *A. parasiticus*, two common AF-producing fungi, frequently infect important food crops such as maize and peanuts, especially when plants are stressed.^1^ AFs can be produced when these fungus-contaminated crops are not sufficiently dried before they are put in storage or when stored under humid conditions.^2^ High doses of AFB_1_, the type of AF produced by *A. flavus* and *A. parasiticus*, can lead to death from aflatoxicosis.^3^ AFB_1_ is also a potent carcinogen: chronic exposure can lead to hepatocellular carcinoma, especially in combination with hepatitis B infection.^1^

AF exposure has been hypothesised to lead to mucosal damage and subsequent nutrient malabsorption and increased intestinal permeability and immunomodulation.^1 4^ A number of observational studies have shown an association between AFB_1_ exposure and reduced linear growth in utero and in infants and young children.^5–8^ An inherent limitation of these observational studies is that they cannot fully exclude confounding by factors such as poverty, which is associated with poor cropping, harvesting, postharvest and storage practices, all of which can contribute to AF contamination of crops.^9^ Recent evidence from Kenya shows that the serum AF levels in poor rural women with the worst socioeconomic background was 4.7 to 7.1 times higher than those in women who were less poor.^10^ Poverty is also associated with low-quality diets and frequent infections in children, both of which are associated with growth retardation.^11^

This study used a randomised controlled design to examine the impact of reducing AF exposure on child linear growth and serum AF levels in Eastern Kenya. More specifically, we hypothesised that reducing exposure to AF-contaminated maize would increase linear growth and reduce serum AFB_1_-lysine adduct level in children under 24 months of age. The Mitigating Aflatoxin Exposure to Improve Child Growth in Eastern Kenya (MAICE) study reported here is the first randomised controlled trial to evaluate the effect of reducing AF exposure on child linear growth.

## Methods

The study protocol was published previously.^12^ A summary of the study methods is provided below.

### Study design

A cluster randomised longitudinal trial design was used. Clusters were defined as villages, typically consisting of 50 to 175 households (mean of 95, median of 86). The study was conducted in rural areas within Meru and Tharaka-Nithi counties in Kenya, an area where maize is the predominant crop and frequent aflatoxicosis outbreaks and widespread contamination of maize have been reported.^13–17^ A total of 56 villages were randomly selected for inclusion in the study.

A detailed data analysis plan was approved by the trial steering committee. Both the published protocol^12^ and the data analysis plan were added to the public trial registry.

### Participants

**Figure 1:**
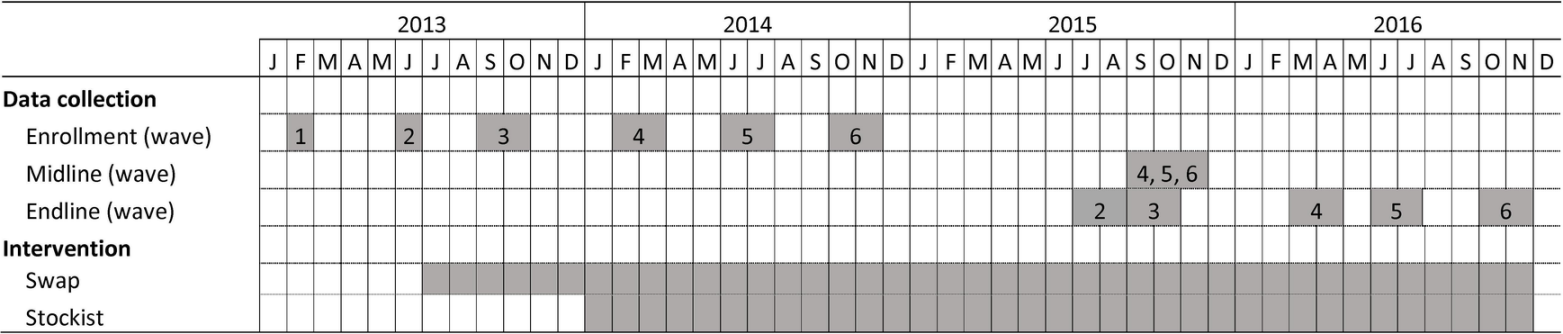
Study timeline.

Enrolment into the study was conducted in six waves, each 4 months apart (figure 1). In each wave, women in the fifth to final month of pregnancy (by the woman’s estimate) were invited to enrol in the study. One newly born child of each pregnant woman became part of the study. In case of twins or triplets, the names of children were ranked alphabetically, and the first was selected to be the study child. The child was followed until 24 months after study enrolment during pregnancy.

### Randomisation and masking

Using Stata, Kelly Jones assigned each village a random number from a uniform [0,1] distribution. Villages with a random number ≤0.5 were assigned to the intervention group; all others were assigned to the control group. Due to the nature of the intervention, assignment could not be masked.

### Procedures

The MAICE intervention consisted of a swapping and a stockist component. In the swapping component, households were offered monthly rapid maize testing at home by trained local staff. If the household agreed, any stored maize that the household was planning to consume over the next 2 months was tested using the US Grain Inspection, Packers and Stockyards Administration (GIPSA) verified Romer AgraStrip rapid test with a 10 parts per billion (ppb) detection threshold, according to manufacturer instructions.^12^ Stored maize found to contain over 10 ppb AF (the Kenyan regulatory limit for AF contamination) was replaced with an equal amount of “safe” maize, that is, maize tested to be under the regulatory limit. This component of the intervention was started in July 2013, shortly, after the second enrolment wave and several months after the originally planned intervention start date due to contracting and logistical delays (figure 1). In the first months of the trial, we observed that households acquired the majority of the maize they consumed through the market and not from own production. This was unexpected (based on the formative study, we conducted with 30 maize farmers in Meru county in 2012) and was due to an unusually poor maize harvest in the study area in 2013. Since household maize purchases were typically small and frequent (more than once per month as originally hypothesised), the swapping component was less effective in reducing household AF exposure than anticipated. To increase intervention effectiveness, the stockist component was rolled out between January and March 2014, that is, after trial commencement. In this component, maize containing less than 10 ppb AF was supplied to at least one shopkeeper in each of the intervention villages; study household were encouraged at an initial village meeting, and monthly during the swapping visits, to purchase this clean maize from the local stockist as needed.

Since wave 1 participants were possibly not exposed to both components of the intervention long enough for it to have an impact (figure 1), a sixth enrolment wave was added to the five originally included in the study design. Per the original protocol, wave 1 households in the MAICE intervention group continued to receive the intervention for a 2-year period, but this group was excluded from follow-up data collection, in line with the registered, and subsequently published, study protocol.^12^

All data collection occurred at study participants’ homes through face-to-face interviews using computer-assisted personal interview software on handheld tablets. The expectant mother was interviewed immediately after enrolment, and her height and weight were measured. A similar survey was repeated at endline, that is, at 24 months, after enrolment of the pregnant mothers. At the time that the endline survey was conducted among the wave 3 participants, a midline survey was conducted among participants enrolled in the fourth through sixth waves, per the published protocol (figure 1). At each follow-up visit (midline for waves 4 through six and endline for all waves), the length and weight of the index child (the child the mother was expecting at enrolment) was measured using standard methods.^18^ A venous blood sample was taken from the child for serum AFB_1_-lysine adduct analysis. In addition to collecting data on the primary and secondary outcomes, data were collected on household demographics, education level of household members, household food insecurity,^19^ household dietary diversity using the Food and Nutrition Technical Assistance (FANTA) Household Dietary Diversity Score,^20^ household asset ownership and maternal height.

### Outcomes

The primary outcomes were child length-for-age Z-score (LAZ, both as a continuous variable and as the prevalence of stunting) and child serum AFB_1_-lysine adduct level at endline (24 months after enrolment of their pregnant mother, when children were on average 22 months old). Child LAZ, the prevalence of stunting and child serum AFB_1_-lysine adduct level at midline (11 to 19 months after enrolment, when average child age was 13.3±3.6 months) were considered secondary outcomes.

Anthropometric Z-scores were calculated using the 2006 WHO growth standards.^21^ Stunting was defined as LAZ below −2 SD of the age and sex-specific median of the growth standard. To determine serum AFB_1_-lysine adduct level, serum samples were analysed using the high-performance liquid chromatography (HPLC)-fluorescence method. Details on the HPLC analytic method used in this study have been published previously.^10^ Serum AFB_1_-lysine adduct level was log transformed for the analyses.

### Statistical analyses

Based on the magnitude of effect sizes of known effective nutrition interventions on linear growth,^22^ the minimum detectable effect (MDE) for exposure to the intervention for the full 24-month period was set to 0.3 LAZ. This was then adjusted downward taking into account, the partial exposure to the intervention of participants recruited in waves 2 and 3 (prior to introduction of the stockist component) to give an MDE of 0.281. Other sample size parameters were a one-sided alpha of 0.05, 80% power and a SD of LAZ of 1.28. We first calculated the required sample size under the assumption of individual randomisation. Next, we adjusted the calculations for an intracluster correlation of 0.05% and 9% attrition, and we assumed that 15% of variation in the outcome would be explained by baseline covariates. The estimated total number of participants required to achieve the study objectives was 924 across 56 villages. This sample size provides sufficient statistical power to detect an effect size of approximately 0.2 on serum AF.

Data analysis followed the statistical analysis plan. All analyses were conducted using Stata V.15.0. In line with the Consolidated Standards of Reporting Trials (CONSORT) 2010 guidelines, no formal comparison of baseline means between the intervention and control arm was conducted.^23^ Intention-to-treat analysis was used to assess the impact of the intervention. The impact on linear growth and serum AF levels at endline was assessed for all enrolment waves (excluding wave 1) combined.

Ordinary least squares regression analysis was used to estimate the impact of the intervention. We estimated models that controlled for the complete set of prespecified baseline covariates, and reduced models, that is, models retaining only those socioeconomic covariates that were statistically significant. At the household level, the covariates included household food insecurity (using FANTA’s Household Food Insecurity Access Scale^19^), household dietary diversity (using FANTA’s Household Dietary Diversity Score^20^), household assets (a simple count of the total number of assets owned, proxy for household wealth) and the number of adult equivalents in the household. At the individual level, the models controlled for child sex and age (in months), maternal age, height and education and the educational level of the head of household. Finally, we controlled for study enrolment wave and birth season (for the linear growth outcomes) and season of measurement (for the serum AF outcome). Following the CONSORT guidelines, we also present impact analyses unadjusted for socioeconomic covariates (these estimates do control for biological covariates such as child age and sex).^23^

Two types of sensitivity analyses were conducted for the primary outcome analyses. To assess the importance of observations lost to follow-up, we estimated the probability of dropout using an augmented set of baseline variables (ie, the covariates included in the impact model and a number of other variables possibly related to loss to follow-up). The predicted linear probability (ie, the predicted log odds) was then added as a covariate to the regression model used to estimate the impact of the intervention. Second, we used multiple imputation to fill in missing values for observations that did not dropout, but had some missing information. Sequential imputation using chained equations (Stata’s mi command) was used. Once values were imputed, the impact regression model was estimated. The standard errors in all analyses were adjusted for the (potential) lack of independence between observations in the same village. A p value of 0.05 was considered statistically significant. Given the clear a priori hypothesis of the trial, and in line with the sample size calculation and data analysis plan, one-sided tests were used to assess the significance of the intervention on all outcomes.^24^

In addition to the analyses to assess the impact of the intervention, we present additional exploratory analyses as online supporting material to help with the interpretation of the findings. These include changes over time in mothers’ serum AFB1-lysine adduct level at enrolment, control group children’s serum AFB1-lysine adduct level at endline and the proportion of households with maize samples above the 10 ppb AF testing threshold.

The trial was registered at American Economic Association's registry for randomised controlled trials as AEARCTR-0000105 on November 6, 2013 and updated in 2014 to reflect the addition of the stockist intervention, addition of the sixth enrolment wave and expansion of the sample size.

### Role of the funding source

The views expressed in this paper are those of the authors and not necessarily those of the funding bodies. The funder of the study had no role in study design, data collection, data analysis, data interpretation or writing of the report. The corresponding author had full access to all the data in the study and had final responsibility for the decision to submit.

## Results

**Figure 2:**
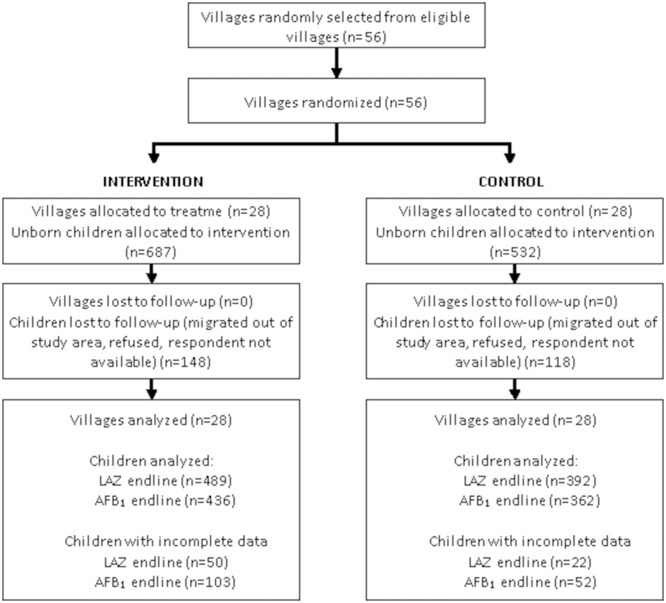
Trial profile. AFB, Alfatoxin B1; LAZ, length-for-age Z-score.

Enrolment exceeded the minimum sample size indicated by power calculations due to higher than expected fertility rates in the study villages (figure 2). This was offset by higher than expected loss to follow-up. Of the 1219 (687 intervention, 532 control) unborn children enrolled in the study, 881 (72%) were included in the LAZ and 798 (65%) in the serum AFB_1_ analysis. A total of 251 (148 intervention, 118 control) were lost to follow-up; an additional 72 children (50 intervention, 22 control) and 155 children (103 intervention, 52 control) had incomplete data for the LAZ and AFB analyses at endline, respectively. The discrepancy in completeness of follow-up data between intervention and control groups was driven by very low rates of interview completion in two relatively large villages where several challenges, including rumours of witchcraft, were recorded by survey staff. The proportion of observations with complete data does not differ significantly (p>0.05) by intervention status for any of the reported outcomes.

Children were on average around 22 months old at endline and 4 out of 10 were stunted (table 1). AFB_1_-lysine adduct was detectable in all analysed endline child serum samples. The mean level of serum AFB_1_-lysine adduct was 18.1 pg/mg albumin (median 6.1 pg/mg albumin). Mothers were about 25 years old and had low levels of schooling. Similar low levels of schooling were found for the head of household. The average child age at midline (limited to waves 4, 5 and 6) was 13 months, and the prevalence of stunting in those children was 23%. Baseline characteristics were well balanced across trial arms (table 1).

**Table 1:** Characteristics of the study population

	Intervention	Control
Mother, baseline (n)	489	392
Height (cm)	157.47±6.01	158.10±6.46
Age (years)	25.74±6.13	25.70±6.24
Education		
None/primary incomplete (%)	54.81	52.3
Primary complete (%)	29.04	30.1
(Some) secondary (%)	16.16	17.6
Ln serum aflatoxin B1 (AFB1)-lysine adduct level	2.69±1.61	2.74±1.68
Head of household education, baseline (n)	489	392
None/primary incomplete (%)	56.85	51.79
Primary complete (%)	27.81	26.79
(Some) secondary (%)	15.34	21.43
Household, baseline (n)	489	392
Food Security Scale		
Food secure (%)	19.02	17.35
Mildly food insecure (%)	14.31	13.52
Moderately food insecure (%)	37.63	36.48
Severely food insecure (%)	29.04	32.65
Dietary diversity score tertile		
First (%)	33.13	36.22
Second (%)	34.56	34.18
Third (%)	32.31	29.59
Assets (low) (%)	63.39	68.62
Adult equivalents	36.61	31.38
Child, endline (n)	489	392
Age endline (months)	22.13±2.28	22.02±2.63
Male (%)	51.12	51.79
Child, midline (n)	363	307
Age midline (months)	13.36±3.73	13.45±3.60

The intervention had no effect on child LAZ (impact estimate −0.01, 95% CI −0.165 to 0.146; one-sided p value 0.551) or on the prevalence of stunting at endline (0.015,–0.51 to 0.036; one-sided p value 0.671) (table 2). The intervention had a significant effect on ln serum AFB_1_-lysine adduct levels at endline: the intervention reduced serum AFB_1_-lysine adduct levels by approximately 27% (–0.273, –0.547 to 0.001; one-sided p value 0.025). A significant effect on child linear growth was found at midline: the intervention increased LAZ by 0.16 SD and reduced the prevalence of stunting by seven percentage points. No impact was found on serum AFB_1_-lysine adduct levels at midline. The estimates unadjusted for socioeconomic baseline variables were similar but less precisely estimated (online supplementary table 2). The sensitivity analyses (reduced model, model including the predicted log odds of dropout, multiple imputation) did not change any of the findings (online supplementary table 3).

10.1136/bmjgh-2018-000983.supp1 Supplementary data

**Table 2:** Impact of the intervention on child LAZ, stunting and ln serum AFB1-lysine adduct levels

	Intervention	Control	Impact estimate (95% CI)	P values*	n
Primary outcomes					
LAZ (endline)	−1.68±1.08	−1.59±1.27	−0.010 (−0.165 to 0.146)	0.551	881
Stunting (endline)	39.26	35.71	0.015 (−0.052 to 0.082)	0.671	881
Ln serum AFB1-lysine adduct level (endline)†	1.78±1.28	2.01±1.37	−0.273 (−0.547 to 0.001)	0.025	798
Secondary outcomes					
LAZ (midline)	−1.17±1.13	−1.32±1.24	0.160 (−0.009 to 0.33)	0.032	613
Stunting (midline)	20.00	26.57	−0.066 (−0.125 to −0.007)	0.015	613
Ln serum AFB1-lysine adduct level (midline)‡	1.54±1.17	1.60±1.21	−0.062 (−0.299 to 0.175)	0.302	524

*One-sided p values are shown.

†Sample sizes for serum aflatoxin were 436 and 362 in the intervention and control arms at endline, respectively. Absolute AFB1-lysine adduct level were 14.79±30.24 pg/mg albumin in the intervention arm and 22.19±58.1 pg/mg albumin in the control arm.

‡Sample sizes for serum aflatoxin were 279 and 245 in the intervention and control arms at midline, respectively. Absolute AFB1-lysine adduct level were 9.83±18.62 pg/mg albumin in the intervention arm and 16.12±71.94 pg/mg albumin in the control arm.

AFB1, aflatoxin B1; LAZ, length-for-age Z-score.

The exploratory analyses online supplementary figure 1 show a spike in maternal serum AFB_1_-lysine adduct levels in June and November 2014, which corresponds to a spike in the proportion of households in treatment areas with stored maize testing above 10 ppb. For the rest of the study period, both the proportion of households with contaminated maize and serum lys-AFB levels are considerably lower. Child serum AFB_1_-lysine adduct levels in the control group were lowest during endline waves 2 to 4.

## Discussion

This study, carried out in Eastern Kenya, is the first cluster randomised controlled trial to test whether AF exposure stunts child linear growth. Reducing AF exposure through a swapping and stockist intervention significantly lowered serum AF levels: at study endline (24 months after study enrolment during pregnancy), children in intervention communities had serum AF levels that were 27% lower than in the control communities. The intervention, however, did not improve child linear growth at this prespecified study endline.

The effect on serum AF indicates that the lack of impact on growth is not a consequence of a failure to reduce AF exposure. In addition, AF exposure levels were similar or higher than those in previous observational studies documenting associations between AF and child linear growth.^6 8 25^ Serum AF levels in pregnant women at enrolment waves 2 to 4 (June 2013 through February 2014) ranged from 3.8 to 8.4 pg/mg albumin (online supplementary figure 1), similar to those reported by Turner *et al* in pregnant women (5.3 pg/mg after adjusting for the factors published by McCoy to account for different analytical methods).^6 25^ During the final two enrolment waves for pregnant women (June and November 2014), however, exposure levels were considerably higher (the geometric mean of AFB_1_-lysine adduct levels were between 35 and 40 pg/mg). Levels of AF detected in the intervention households’ stored maize followed a similar pattern. From September 2013 to May 2014, a period that spans the first three enrolment waves included in the study, the mean proportion of households who had maize in store that tested over 10 ppb (the Kenyan regulatory threshold) was 4.6% (N=1163), and the geometric mean contamination in a subsample of those exceeding 10 ppb was 36.7 ppb (N=48). From June to November 2014, when the last two waves were enrolled, 19.5% of maize samples tested above 10 ppb (N=1668), and the geometric mean among a random subset from those over 10 ppb was 563 ppb (N=51). This is much higher than levels typically observed in studies documenting crop AF prevalence.^26 27^ The levels in children at endline (4.5 to 8.3 pg/mg, August 2015 through October 2016) were comparable to those reported by Gong *et al* in children 16 to 37 months of age (geometric mean 4.3 pg/mg, after adjustment using McCoy *et al*).^8 25^

A strength of our study is its design: it is the first to use a randomised trial to study the effects of reducing AF exposure. A key limitation (inherent to this type of study) is the different nature of our two primary outcomes: the effect of environmental insults on linear growth is cumulative and takes many months (or years) to become observable. Serum AFB_1_-lysine adduct reflects exposure to AF in the past 3 months. The different response time of the two outcome variables and the known seasonal variation in AF exposure online supplementary figure 1 may explain the apparent paradox in the findings at endline (reduction in serum AF levels; no effect on growth) and midline (no effect on serum levels; effect on growth). By-wave analysis of the impact of the intervention on serum AF levels (online supplementary table 4) shows that the impact of the intervention was limited to endline waves 5 and 6, when serum AF was highest in the control group children. This suggests that AF exposure during the other waves of data collection may have been too low for a reduction to be detected.

As a consequence of the difference in response time and seasonal variation, linking (reductions in) exposure to (improvements in) linear growth is challenging. Ideally, this would entail quarterly monitoring of serum AFB_1_-lysine adduct, which was cost prohibitive for this study. The biological pathways that might explain the effect of AF exposure on linear growth are poorly understood: environmental enteric dysfunction, immunomodulation and changes in the hepatic metabolism of micronutrients have been proposed as possible mechanisms.^4 28^ We did not collect biomarkers for any of these. Another limitation is the loss to follow-up in the study, which was relatively high. Our sensitivity analyses, however, do not suggest that this changed our results.

The midline growth effect (secondary outcome) leads to important questions regarding the toxicity of AF for growth. The intervention appears to have had a large positive growth effect when these children were on average around 13 months old. No effect was found on serum AF levels at midline, which were relatively low in control group children at this time. The growth effect, however, was no longer observed in the same children (ie, children enrolled in waves 4, 5 and 6) at endline. To ensure that this finding was not due to differential dropout between midline and endline, we re-estimated the growth effects on a homogenous sample of children present at both time points and found that the results did not change (online supplementary table 5). The large effect at midline combined with the absence of an effect at endline indicate that the toxic effect of AF might vary by age. Our findings suggest that protection from the 2014 AF spike early in life (ie, in utero or at very young ages, as was the case for wave 4, 5 and 6 children) was particularly beneficial (online supplementary figure 1). The disappearance of the impact between midline and endline could indicate that the relative (biological) importance of AF exposure as an inhibitor of linear growth decreases as children grow older. Larger reductions in AF exposure may also have been needed to result in a (continued) effect on linear growth. In addition, children’s nutrient intake between midline and endline might have been too deficient to sustain the growth advantage the children had accumulated by midline. These factors may have resulted in children regressing to the status of the children in the control group. Unfortunately, we do not have the necessary data to further explore these hypothesised pathways. Finally, the disappearance of the linear growth impact does not imply that the intervention did not confer other potentially permanent benefits to the children in the intervention group, such as improvements in neurocognitive development and immune function.

The study confirms that exposure to AF can be substantially reduced through interventions (supplemental text on the uptake of the intervention). Given the complexity and cost of organising regular swapping and the stockist approach, the intervention tested here is not feasible for implementation at scale. Other solutions, however, are available. A postharvest intervention was shown to substantially reduce serum AF levels in Guinea.^26^ Aflasafe, which inoculates the soil with non-toxicogenic *Aspergillus* strains, can be used in large scale programme.^29^

Our findings have implications for future studies and public health. First, as found in this study, AF exposure varies considerably over space and time. This means that the potential benefit of interventions aimed at reducing AF exposure is to a large extent outside the control of the researchers. Studies in multiple locations, during different seasons and for varying time periods will thus be needed to answer the AF-stunting question definitively. Second, our findings suggest that AF’s effect on growth may vary with age. Future studies should explore these age-varying effects. Third, even if future studies find no effect of AF on linear growth, research should focus on better understanding the (hypothesised) effects of AF exposure on environmental enteric dysfunction, systemic inflammation, immunomodulation and changes in the hepatic metabolism of micronutrients.^4 28^ Several of these pathways are likely to affect other critical outcomes, such as child health and development.

Finally, it must be noted that there are multiple benefits to AF control, which include agricultural productivity, access to domestic and export markets and health. Decisions about AF control should assess the joint benefits and not be limited to the potential effect on child linear growth.^1 30 31^
