# Correction: *The impact of reducing dietary aflatoxin exposure on child linear growth: a cluster randomized controlled trial in Kenya*

**DOI:** 10.1136/bmjgh-2018-000983corr1

**Published:** 2019-02-02

Hoffmann V, Jones K, Leroy JL. The impact of reducing dietary aflatoxin exposure on child linear growth: a cluster randomised controlled trial in Kenya. *BMJ Global Health* 2018;3:e000983. doi: 10.1136/bmjgh-2018-000983

The authors want to alert the readers on the corrected Funding and Acknowledgment statements.

**Funding**

This research was funded by UK aid from the British people and the CGIAR Research Programme on Agriculture for Nutrition and Health (A4NH).

**Acknowledgements**

We thank Celeste Sununtnasuk and Alexia Pretari for providing excellent research support and Jia-Sheng Wang for analysing the serum samples. The study would not have been possible with the outstanding field coordination by Nouhoum Traore and Lulu Tian. We thank the members of the Trial Steering Committee (Rebecca Stoltzfus, Paul Turner and Edward Frongillo) for their very useful guidance and advice.

