# Supplementary material for: The impact of reducing dietary aflatoxin exposure on child linear growth: a cluster randomised controlled trial in Kenya
Source: BMJ Glob Health. 2018 Dec 1;3(6):e000983. doi: 10.1136/bmjgh-2018-000983 (PMC6278920; doi:10.1136/bmjgh-2018-000983)

**Supplementary material**

**Supplemental text**

**Intervention uptake -** Data collected during swapping visits indicates that treatment households obtained on average 47% of their maize grains (out of the total monthly average purchase of 18.4 kg) and 78% of their maize flour (out of the total monthly average purchase of 4.15 kg) from a study stockist. Nearly all households (98%) in treatment areas had their stored maize tested at least once, and the mean (median) number of times maize was tested was 5.2 (5). About half of all treatment households (54%) had their maize swapped at least once; among these, maize was swapped an average of 1.6 times.

**Supplemental Table 1: Intracluster correlation for outcome variables**

| | Intracluster correlation (95% CI) |
|---|---|
| Primary outcomes | |
| LAZ (endline) | 0.07 (0.01 to 0.13) |
| Stunting (endline) | 0.03 (0.00 to 0.07) |
| Ln serum AFB1-lysine adduct level (endline)[b] | 0.14 (0.05 to 0.22) |
| Secondary outcomes | |
| LAZ (midline) | 0.03 (0.00 to 0.08) |
| Stunting (midline) | 0.03 (0.00 to 0.08) |
| Ln serum AFB1-lysine adduct level (midline) | 0.06 (0.00 to 0.12) |

**Supplemental Table 2: Impact of the intervention on child LAZ, stunting, and Ln serum AFB1-lysine adduct levels (analyses not controlling for socio-economic baseline variables)**

| | Intervention | Control | Impact estimate (95% CI) | P-value[a] | n |
|---|---|---|---|---|---|
| Primary outcomes | | | | | |
| LAZ (endline) | -1.68±1.08 | -1.59±1.27 | -0.017 (-0.199 to 0.164) | 0.575 | 881 |
| Stunting (endline) | 39.26 | 35.71 | 0.018 (-0.054 to 0.090) | 0.694 | 881 |
| Ln serum AFB1-lysine adduct level (endline)[b] | 1.78±1.28 | 2.01±1.37 | -0.235 (-0.575 to 0.105) | 0.086 | 798 |
| Secondary outcomes | | | | | |
| LAZ (midline) | -1.17±1.13 | -1.32±1.24 | 0.148 (-0.040 to 0.337) | 0.060 | 613 |
| Stunting (midline) | 20.00 | 26.57 | -0.067 (-0.133 to -0.001) | 0.024 | 613 |
| Ln serum AFB1-lysine adduct level (midline)[c] | 1.54±1.17 | 1.60±1.21 | -0.039 (-0.312 to 0.243) | 0.390 | 524 |

[a] One-sided p-values are shown.

[b] Sample sizes for serum aflatoxin were 436 and 362 in the intervention and control arms at endline, respectively. Absolute AFB1-lysine adduct level were 14.79±30.24 pg /mg albumin in the intervention arm and 22.19±58.1 pg /mg albumin in the control arm.

[c] Sample sizes for serum aflatoxin were 279 and 245 in the intervention and control arms at midline, respectively. Absolute AFB1-lysine adduct level were 9.83±18.62 pg /mg albumin in the intervention arm and 16.12±71.94 pg /mg albumin in the control arm.

**Supplemental Table 3: Sensitivity analyses of the impact of the intervention on child LAZ, stunting, and Ln serum AFB1-lysine adduct levels**

| | Impact estimate (95% CI) | P-value[a] | n |
|---|---|---|---|
| Reduced model | | | |
| LAZ (endline) | -0·029 (-0·19 to 0·132) | 0·639 | 881 |
| Stunting (endline) | 0·025 (-0·045 to 0·094) | 0·758 | 881 |
| Ln serum AFB1-lysine adduct levels (endline) | -0·261 (-0·545 to 0·024) | 0·036 | 798 |
| Logodds model | | | |
| LAZ (endline) | 0·019 (-0·143 to 0·182) | 0·406 | 857 |
| Stunting (endline) | 0·009 (-0·061 to 0·078) | 0·599 | 857 |
| Ln serum AFB1-lysine adduct levels (endline) | -0·279 (-0·546 to -0·011) | 0·021 | 779 |
| Multiple imputation | | | |
| LAZ (endline) | -0·026 (-0·181 to 0·13) | 0·629 | 953 |
| Stunting (endline) | 0·027 (-0·042 to 0·096) | 0·784 | 953 |
| Ln serum AFB1-lysine adduct levels (endline) | -0·254 (-0·516 to 0·008) | 0·028 | 953 |

[a] One-sided p-values are shown.

**Supplemental Table 4: Wave-specific impact of the intervention on child Ln serum AFB1-lysine adduct levels**

| | Impact estimate (95% CI) | P-value[a] | n |
|---|---|---|---|
| Wave 2 | -0.043 (-0.829 to 0.744) | 0.913 | 102 |
| Wave 3 | 0.015 (-0.586 to 0.615) | 0.960 | 88 |
| Wave 4 | -0.029 (-0.586 to 0.527) | 0.542 | 190 |
| Wave 5 | -0.556 (-0.941 to -0.171) | 0.006 | 206 |
| Wave 6 | -0.476 (-0.867 to -0.086) | 0.018 | 212 |

[a] One-sided p-values are shown.

**Supplemental Table 5: Impact of the intervention on child LAZ, stunting, and Ln serum AFB1-lysine adduct levels on a homogenous group of children at endline and midline**

| | Impact estimate (95% CI) | P-value[a] | n |
|---|---|---|---|
| Endline | | | |
| LAZ (endline) | 0·068 (-0·073 to 0·209) | 0·170 | 611 |
| Stunting (endline) | -0·019 (-0·094 to 0·055) | 0·305 | 611 |
| Midline | | | |
| LAZ (endline) | 0·162 (-0·006 to 0·33) | 0·029 | 611 |
| Stunting (endline) | -0·067 (-0·126 to -0·009) | 0·013 | 611 |

[a] One-sided p-values are shown.

**Supplemental Figure 1**

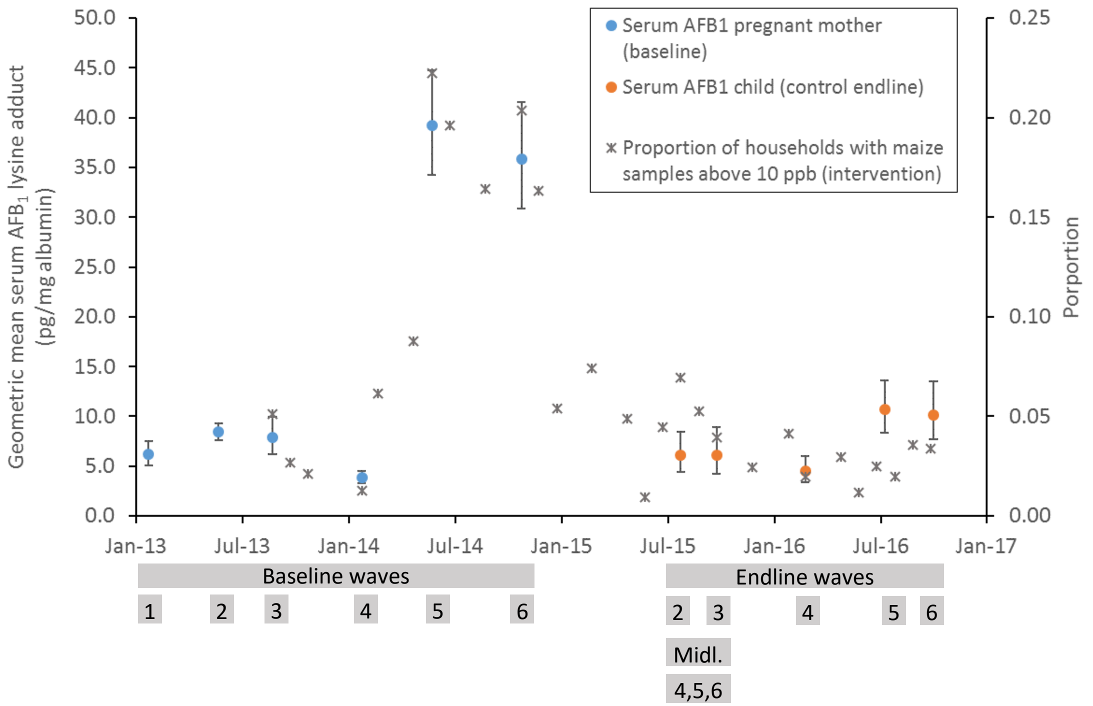

Supplement: Supplementary data [file bmjgh-2018-000983supp001.pdf]
